# Identification of a novel and heterozygous *LMF1* nonsense mutation in an acute pancreatitis patient with severe hypertriglyceridemia, severe obesity and heavy smoking

**DOI:** 10.1186/s12944-019-1012-9

**Published:** 2019-03-18

**Authors:** Wei-Wei Chen, Qi Yang, Xiao-Yao Li, Xiao-Lei Shi, Na Pu, Guo-Tao Lu, Zhi-Hui Tong, Jian-Min Chen, Wei-Qin Li

**Affiliations:** 1 Surgical Intensive Care Unit (SICU), Department of General Surgery, Jinling Hospital, Medical School of Nanjing University, Nanjing, 210000 Jiangsu China; 2 Department of Gastroenterology, Clinical Medical College, Yangzhou University, Yangzhou, 225000 Jiangsu China; 3 EFS, Univ Brest, Inserm, UMR 1078, GGB, F-29200 Brest, France

**Keywords:** Hypertriglyceridemia, Acute pancreatitis, Lipase maturation factor 1, Mutation

## Abstract

**Background:**

Hypertriglyceridemia (HTG) is one of the most common etiologies of acute pancreatitis (AP). Variants in five genes involved in the regulation of plasma lipid metabolism, namely *LPL*, *APOA5*, *APOC2*, *GPIHBP1* and *LMF1*, have been frequently reported to cause or predispose to HTG.

**Methods:**

A Han Chinese patient with HTG-induced AP was assessed for genetic variants by Sanger sequencing of the entire coding and flanking sequences of the above five genes.

**Results:**

The patient was a 32-year-old man with severe obesity (Body Mass Index = 35) and heavy smoking (ten cigarettes per day for more than ten years). At the onset of AP, his serum triglyceride concentration was elevated to 1450.52 mg/dL. We sequenced the entire coding and flanking sequences of the *LPL*, *APOC2*, *APOA5*, *GBIHBP1* and *LMF1* genes in the patient. We found no putative deleterious variants, with the exception of a novel and heterozygous nonsense variant, c.1024C > T (p.Arg342*; rs776584760), in exon 7 of the *LMF1* gene.

**Conclusions:**

This is the first time that a heterozygous *LMF1* nonsense variant was found in a HTG-AP patient with severe obesity and heavy smoking, highlighting an important interplay between genetic and lifestyle factors in the etiology of HTG.

**Electronic supplementary material:**

The online version of this article (10.1186/s12944-019-1012-9) contains supplementary material, which is available to authorized users.

## Background

Hypertriglyceridemia-induced acute pancreatitis (HTG-AP) usually refers to acute pancreatitis (AP) occurred in subjects with fasting serum triglyceride (TG) levels of > 1000 mg/dL (> 11.3 mmol/L) or > 500 mg/dL (> 5.65 mmol/L) with coincidentally detected milky serum and in the absence of other etiologic factors. Hypertriglyceridemia (HTG) is responsible for 2–5% of AP patients in Western countries [1] but the corresponding figure is 7.8–25.6% and tends to increase in China [2–4], demonstrating a marked ethnic difference in the etiology of AP. HTG-AP is more often associated with greater severity and higher recurrence and complication rates as compared with other etiologies of AP. This is particularly true in patients with primary severe HTG.

Variants in five genes involved in the regulation of plasma lipid metabolism, namely *LPL* (encoding lipoprotein lipase, which catalyzes hydrolysis of TG-rich lipoproteins [5]), *APOA5* (encoding apolipoprotein A-V, which stabilizes the lipoprotein–LPL complex [6]), *APOC2* (encoding apolipoprotein C-II, which acts as an essential LPL activator [7]), *GPIHBP1* (encoding glycosylphosphatidylinositol-anchored high density lipoprotein-binding protein 1, which mediates the transmembrane transport and binding of LPL [8]), and *LMF1* (encoding lipase maturation factor 1, which is involved in the folding and expression of LPL [9]), have been frequently reported to cause or predispose to HTG. HTG may be of monogenic origin in some patients, for example, those with the autosomal recessive inherited familial hyperchylomicronemia syndrome. However, the etiology of HTG in most cases is complex and likely involves gene-gene and/or gene-lifestyle interactions [10].

Herein, we describe a case with HTG-AP that appeared to be predisposed by a combination of genetic susceptibility and exposure to harmful lifestyle factors.

## Materials and methods

### Patient

The patient was a 32-year-old man with severe obesity (Body Mass Index (BMI) = 35) and heavy smoking (ten cigarettes per day for more than ten years). He was diagnosed to have moderate HTG (fasting serum TG concentration, ~ 500 mg/dL (~ 5.65 mmol/L)) three years ago (at the age of 29) but had not taken lipid-lowering drugs. His father had diabetes mellitus, his mother was healthy, and there was no known history of HTG in the family.

He was hospitalized elsewhere due to severe abdominal pain associated with nausea and vomiting on June 5, 2017. He was transferred to our Surgical Intensive Care Unit for further treatment on June 11, 2017. The study was performed in accordance with the Helsinki Declaration and was approved by the Ethics Committee of Jingling Hospital, Nanjing University. Bio-samples were obtained after written informed consent.

### Plasma lipid profile analysis

Blood samples were collected after fasting for 12 h. Serum glucose (GLU), TG, total cholesterol (TC), high-density lipoprotein (HDL), low density lipoprotein (LDL), apolipoprotein A1 (APOA1), and apolipoprotein B (APOB) levels were measured enzymatically on an automatic analyzer (Hitachi High-Tech, 7600–120, Japan).

### Mutational analysis of five HTG genes

Genomic DNA was extracted from the peripheral blood cells using a Gentra Puregene Blood kit (Qiagen, Dusseldorf, Germany) according to the manufacturer’s instructions. The entire coding and flanking sequences of *LPL*, *APOA5*, *APOC2*, *LMF1*, and *GPIHBP1* were amplified by polymerase chain reaction (PCR) and subsequently sequenced using both forward and reverse primers (primer sequences available upon email request). Identified mutations were subjected to confirmation by independent PCR amplification and sequencing. Nomenclature for the description of the reported *LMF1* mutation followed the Human Genome Variation Society (HGVS) recommendations [11]. GenBank accession number NM_022773.3 was used as the *LMF1* mRNA reference sequence.

## Results

### Clinical features of the HTG-AP patient

**Fig. 1:**
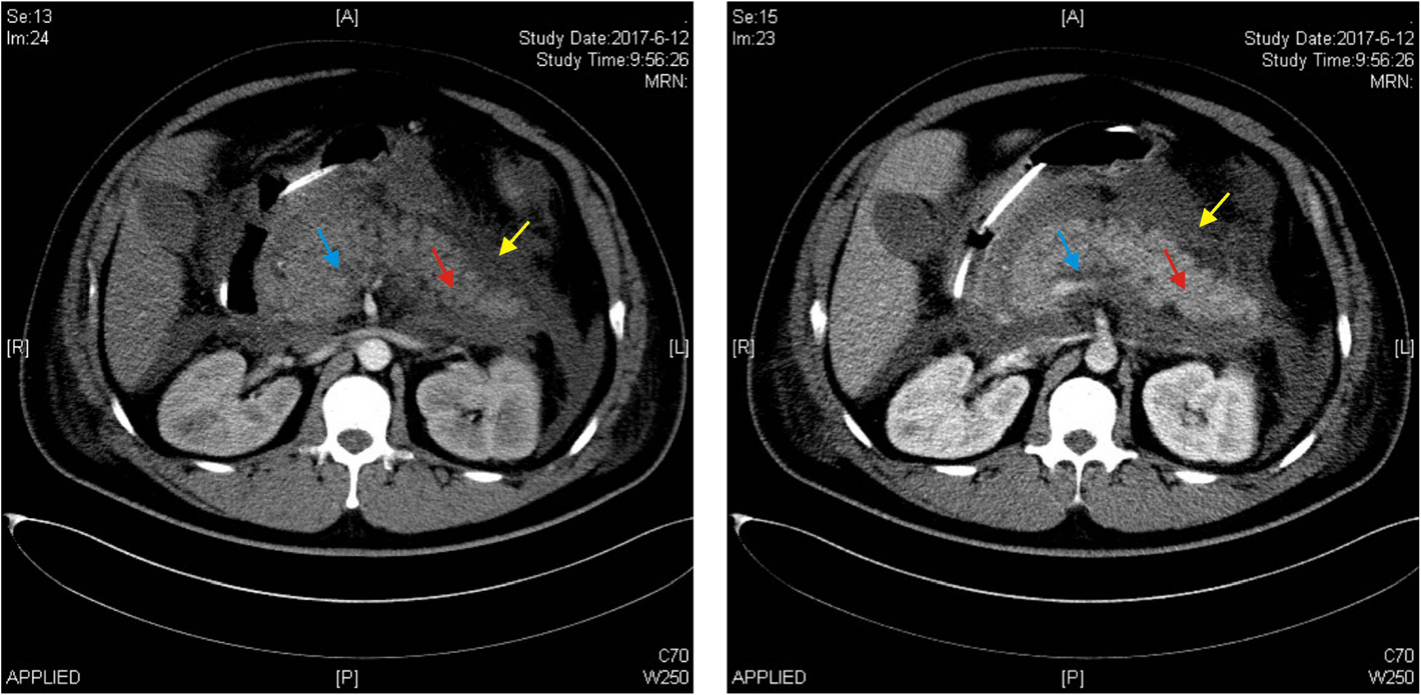
Abdominal computed tomography (CT) showing the enlarged pancreas with adjacent water density shadow, disappearance of the space among peripancreatic adipose tissues in the patient. Left panel, plain CT scan. Right panel, enhanced CT scan. Blue, red and yellow arrowheads indicate acute necrotic collection, pancreatic edema and acute peripancreatic fluid collection, respectively

When the patient was initially hospitalized elsewhere, his blood sample was milky, with the serum TG concentration being elevated to 1450.52 mg/dL (16.39 mmol/L). Moreover, his serum TC and GLU levels were elevated to 6.55 mmol/L and 20.35 mmol/L, respectively. The patient was diagnosed with severe AP with the complication of acute necrotic collection (Fig. 1) according to the 2012 Atlanta consensus report [12]. On admission in our hospital, physical examination of the patient revealed epigastric tenderness and distension without rebound tenderness or Murphy’s sign. Laboratory examination revealed elevations in amylase (274 U/L), white blood cell count (14.5 × 10^9^/L) as well as inflammation markers C-reactive protein (CRP, 156.9 mg/L) and procalcitonin (PCT, 0.821 μg/L). Arterial blood gas analysis showed that pondus hydrogenii (PH) was 7.382 mmol/L, partial pressure of oxygen (PaO_2_) was 86.8 mmol/L, and fraction of inspiration oxygen (FiO_2_) was 33%. Renal and cardiovascular functions were normal.

**Fig. 2:**
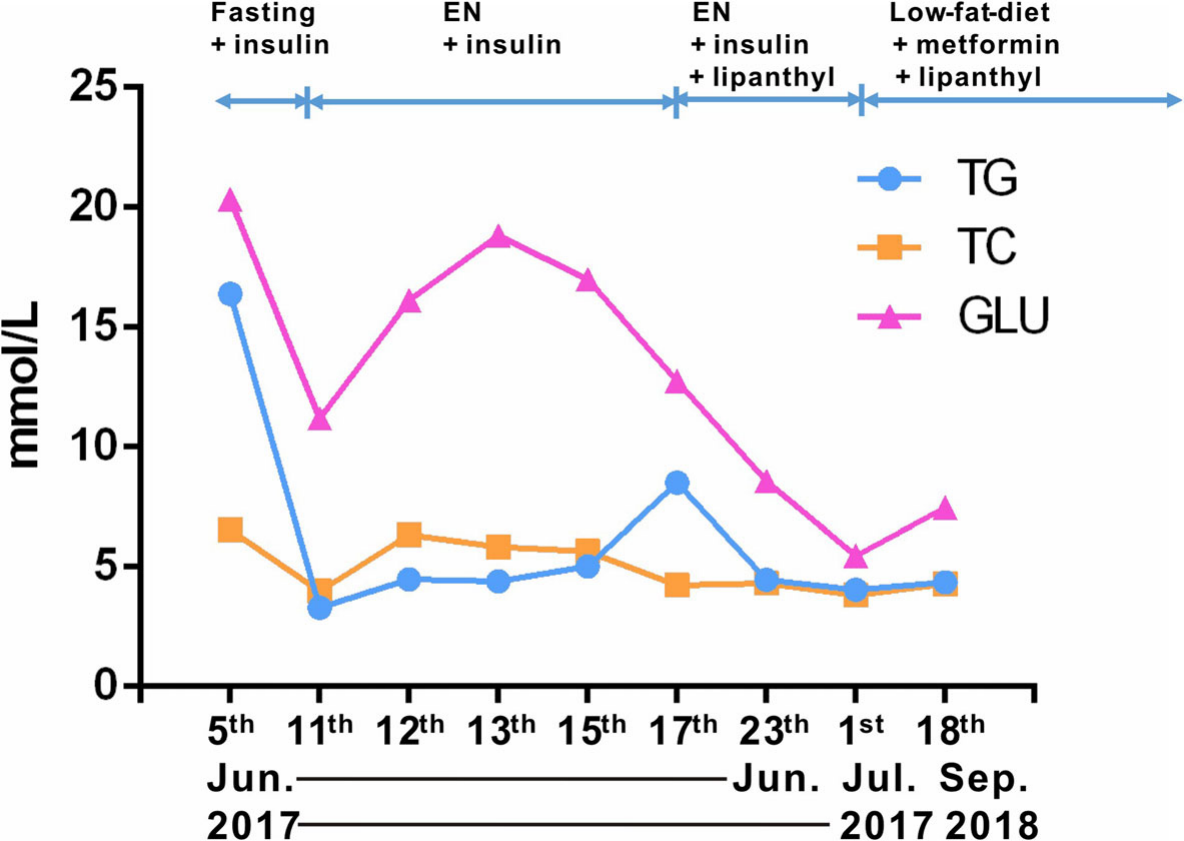
Flow-chart showing treatment procedure and serum TG, TC, and GLU levels in the patient. TG, triglyceride; TC, total cholesterol; GLU, glucose; EN, enteral nutrition

The patient was treated as illustrated in Fig. 2, in which the serum TG, TC, and GLU levels in different time points were also provided. Of particular note, after fasting for 6 days, the patient’s serum TG level plunged to 292.04 mg/dL (3.3 mmol/L). However, his TG level rose to 752.21 mg/dL (8.5 mmol/L) several days after general treatment and enteral nutrition intake. Consequently, he was given lipid-lowering drug Lipanthyl and his TG level decreased to 395.58 mg/dL (4.47 mmol/L). Before discharge on July 1, 2017, the patient’s TG level was 359.29 mg/dL (4.06 mmol/L). Thereafter, he was instructed to adhere to a low fat diet with prescription of Lipanthyl and metformin. A follow-up examination performed in September 2018 revealed a serum TG concentration of 353.98 mg/dL (4.0 mmol/L) (Fig. 2).

### Genetic findings

**Fig. 3:**
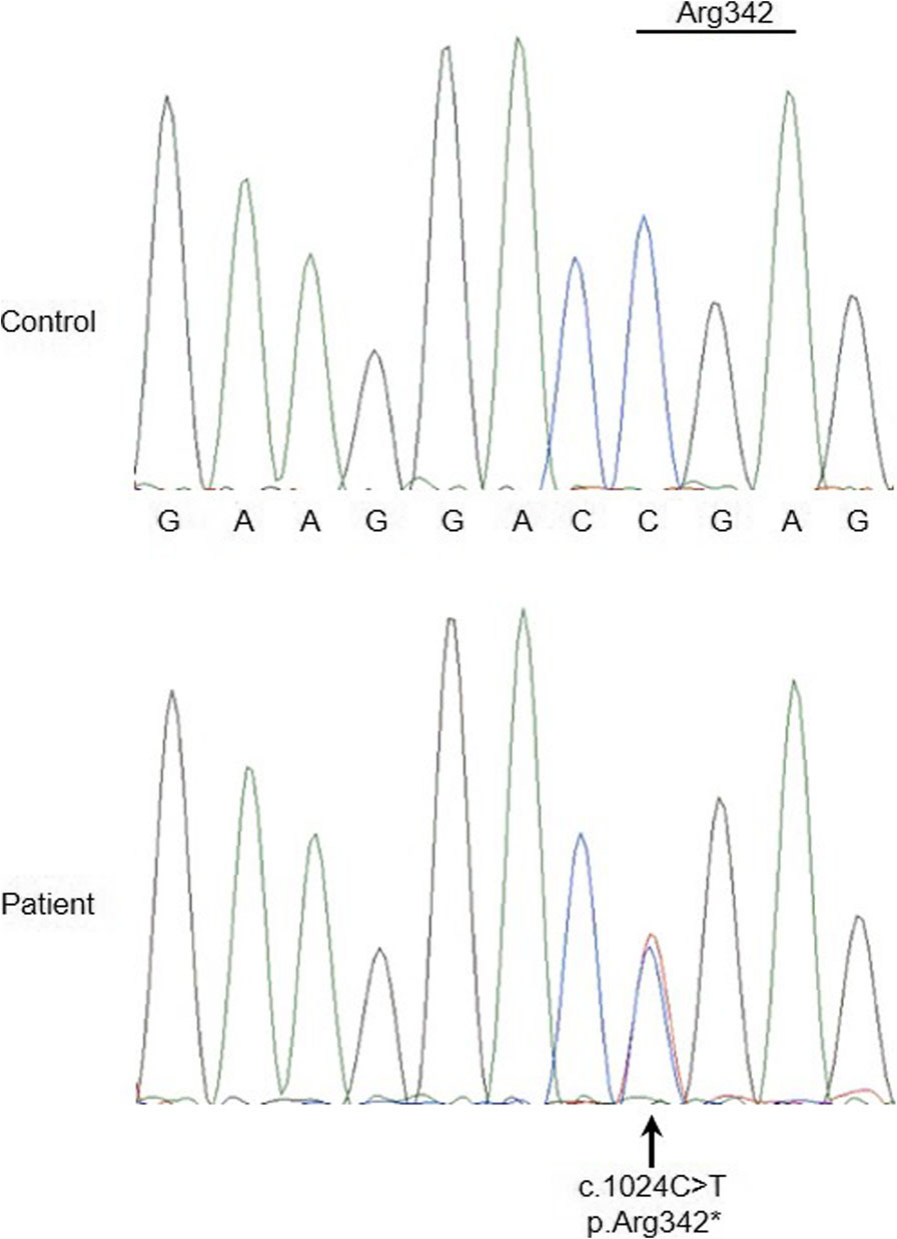
Identification of a novel and heterozygous nonsense variant in the *LMF1* gene. Sequencing electropherograms of the wild-type sequence from a healthy control and the heterozygous *LMF1* c.1024C > T (p.Arg342*) variant from the patient are shown. The affected codon 342 (in exon 7 of the *LMF1* gene) is underlined in the context of the wild-type sequence

We sequenced the entire coding and flanking sequences of the *LPL*, *APOC2*, *APOA5*, *GBIHBP1* and *LMF1* genes in the patient (sequencing data available in Additional file 1). We found no putative deleterious variants, with the exception of a heterozygous nonsense variant, c.1024 C > T (p.Arg342*; rs776584760), in exon 7 of the *LMF1* gene (Fig. 3). This variant was absent in our other 131 HTG-AP patients, 85 AP patients without HTG, and 43 healthy controls (all subjects were Han Chinese). Unfortunately, no family members were available for genetic analysis.

## Discussion

In this study, we identified a heterozygous *LMF1* nonsense mutation, c.1024C > T (p.Arg342*), in a Han Chinese patient with HTG-AP. To our best knowledge, this variant has not been previously reported to be associated with HTG in the literature. Moreover, this variant was also absent in the ClinVar database (https://www.ncbi.nlm.nih.gov/clinvar/; as of November 8, 2018). Further, survey of the Genome Aggregation Database (genomAD, http://gnomad.broadinstitute.org/; as of November 8, 2018) revealed that the c.1024 C > T variant was present at an average allele frequency of 0.00002857 (7 of 244,974) in all populations combined; and its allele frequency in the East Asian population was 0.0002238 (4 of 17,876). In short, the *LMF1* c.1024 C > T variant is extremely rare in normal populations.

The human *LMF1* gene encodes a 567 amino acid protein. Recently, Serveaux Dancer and colleagues assessed the functionality of two *LMF1* nonsense variants, p.Tyr439* and p.Trp464*, by means of an in vitro assay. Specifically, they co-transfected HEK293T cells with the wild-type *LPL* expression vector and the *LMF1* mutant expression vector of interest; and then measured the LPL activity in the culture media of the co-transfected HEK-293 T cells. As such, Serveaux Dancer and colleagues found that both the *LMF1* p.Tyr439* and p.Trp464* nonsense variants caused an almost complete loss of the LPL activity [13]. The *LMF1* c.1024C > T (p.Arg342*) variant reported here, which removes ~ 100 more residues from the C terminus of the protein as compared to p.Tyr439* and p.Trp464*, can be reasonably concluded to cause a complete functional loss of the affected *LMF1* allele.

A particularly interesting finding of the Serveaux Dancer study is that in one of their subjects with severe HTG, their mutational analysis of the *LPL*, *APOC2*, *APOA5*, *GBIHBP1* and *LMF1* genes identified only a single and heterozygous deleterious variant, *LMF1* p.Trp464* (N.B. one of the aforementioned loss-of-function *LMF1* nonsense mutations) [13]. Prior to their report, all heterozygous carriers of nonsense *LMF1* variants had been described to have either normal or at most borderline HTG. Thus, our patient represents the second heterozygous carrier of a *LMF1* nonsense variant who had severe HTG. This notwithstanding, it is pertinent to emphasize an important difference between the two relevant severe HTG patients in terms of their underlying etiological factors. In the Serveaux Dancer study, the two other heterozygous carriers of the *LMF1* p.Trp464* variant had normal TG concentration; and the 8-month-old proband with the heterozygous *LMF1* p.Trp464* variant exhibited severe HTG (1860 mg/dL (21 mmol/L)) on an episode of severe acute gastroenteritis (a condition known to be associated with moderate HTG) without AP. In our study, we were not able to analyze the patient’s parents but neither of them was known to have HTG. Notably, our patient had two harmful lifestyle factors, severe obesity and heavy tobacco abuse. Obesity, producing more TG, is not only associated with primary HTG [14] but also is a risk factor for secondary HTG [15]. Tobacco can influence the lipometabolism of liver, thereby increasing free fatty acid delivery into plasma that can in turn triggers a rise in TG level due to the defective lipolytic system [16]. In addition, tobacco has also been reported to be a risk factor for AP development [17]. In short, whereas the two severe HTG patients had *LMF1* variants of similar functional effects, they had quite different secondary HTG-predisposing factors.

Finally, we would like to make two points. First, the LMF1 protein expression level in the patient carrying the heterozygous *LMF1* nonsense variant, c.1024C > T (p.Arg342*), is assumed to be half of that in health controls. We were not able to confirm this assumption due to the unavailability of pathophysiologically relevant tissue(s) from the patient. Second, “LPL is also expressed at high levels in certain regions of the brain. Why LPL is produced in neurons of the brain remains an enigma ….” [18]. Intriguingly, LMF1, which is involved in the folding and expression of LPL, is also highly expressed in the brain (see https://www.proteinatlas.org/search/lmf1).

## Conclusions

In conclusion, we have for the first time discovered a novel and heterozygous *LMF1* nonsense variant in a HTG-AP patient with severe obesity and heavy smoking, highlighting an important interplay between genetic and lifestyle factors in the etiology of HTG.

## Additional file

Additional file 1: Sequencing data. (DOCX 22 kb)

## Abbreviations

AP: Acute pancreatitis
APOA1: Apo-protein A1
APOA5: Apolipoprotein A-V
APOB: Apo-protein B
APOC2: Apolipoprotein C-II
BMI: Body mass index
CRP: C-reactive protein
EN: Enteral nutrition
FiO_2_: fraction of inspiration oxygen
Glu: Glucose
GPIHBP1: Glycosylphosphatidylinositol-anchored high-density lipoprotein binding protein 1
HDL: High density lipoprotein
HTG: Hypertriglyceridemia
LDL: Low density lipoprotein
LMF1: Lipase maturation factor 1
LPL: Lipoprotein lipase
PaO_2_: Partial pressure of oxygen
PCR: Polymerase chain reaction
PCT: Procalcitonin
PH: Pondus hydrogenii
TC: Total cholesterol
TG: Triglyceridemia
