# Supplementary material for: Identification of a novel and heterozygous LMF1 nonsense mutation in an acute pancreatitis patient with severe hypertriglyceridemia, severe obesity and heavy smoking
Source: Lipids Health Dis. 2019 Mar 18;18:68. doi: 10.1186/s12944-019-1012-9 (PMC6421687; doi:10.1186/s12944-019-1012-9)
Supplement: Supplementary file 1 — Sequencing data. (DOCX 22 kb) [file 12944_2019_1012_MOESM1_ESM.docx]

**Supplementary Information**

Sequencing data with respect to the entire coding and flanking sequences of the *LPL*, *APOC2*, *APOA5*, *GBIHBP1* and *LMF1* genes analyzed in the patient. Coding sequences are in upper-case letters whilst flanking sequences are in lower-case letters. All sequences are from 5’ to 3’. Variants found in the patient are highlighted in blue, with their corresponding nomenclatures and minor allele frequencies (in the East Asian population in accordance with genomAD) being also provided.

***LMF1*（NM_022773.3）**

**>Exon 1**

gagtgcgccctccccgcacatgcgccctgacagcccaacaATGGCGGCGCCCGCGGAGTCGCTGAGGAGGCGGAAGACTGGGTACTCGGATCCGGAGCCTGAGTCGCCGCCCGCGCCGGGGCGTGGCCCCGCAGGCTCTCCGGCCCATCTCCACACGGGCACCTTCTGGCTGACCCGGATCGTGCTCCTGAAGGCCCTAGCCTTCGTGTACTgtgagtgccgggcgggcccgggacatccgccagtgtcacctccgcgcaccacgacccccgccctccctcgagactcctccgcg

**>Exon 2**

gcctgcctcgccccgcattctgagctgcgcccatggtgtccacatgtggtttgttcctagtcatgcttgttttgtcttctttttcttccagTCGTGGCATTCCTGGTGGCTTTCCATCAGAACAAGCAGCTCATCGGTGACAGGGGGCTGCTTCCCTGCAGAGTGTTCCTGAAGAACTTCCAGCAGTACTTCCAGGACAGGAC(G/A)AGCTGGGAAGTCTTCAGCTACATGCCCACCATCCTCTGGCTGATGGACTGGTCAGACATGAACTCCAACCTGGACTTGCTGGCTCTTCTCGGACTGGGCATCTCGTCTTTCGTACTGATCACGGGCTGCGCCAACATGCTTCTCATGGCTGCCCTGTGGGGCCTCTACATGTCCCTGGTTAATGTGGGCCATGTCTGgtgagtagcaggaatggggcggtcggagcttagggcttgctgtcactcagcacaggcactgcaggtgtcctggcacttggaaagactggacgagcgtttatcttaaattccttcaggagggtattgtcattttacttaggccatggcagagcggccagtgcaccagccacagcagc

c.306G>A (p.Thr102=); a common and synonymous variant in the East Asian population (minor allele frequency, 40%).

**>Exon 3**

atgcccagcgggcaggtcctcctcgcagggcttccctgaccccacaccgtgctgggtgccagggtgggcttcactcagtggggaaacagtgggtttttggttgaaacaagccaaagtgttaatactcgtttcttcttttgtttttcagGTACTCTTTCGgtaagtgagatgcatttagagaaagctctgcgagagttgagcgttgtgtgtttggtatgtatcttcacgcacttttcctggcctctccccttccttagcctctgccatcagccttcccagtatgcacggagggca

**>Exon 4**

ggctggtgtctctcagtagcaagatcagccctgtctcaaggaccgtttccattaaactgagtttctgaaatgatttctttgtgaaatgtgtggcttccattaactcacctttggtatgtctcgttgttttagGATGGGAGTCCCAGCTTCTGGAGAC(G/A)GG(G/A)TTCCTGGGGATCTTCCTGTGCCCTCTGTGGACGCTGTCAAGGCTGCCCCAGCATACCCCCACATCCCGGATTGTCCTGTGGGGCTTCCGGTGGCTGATCTTCAGGATCATGCTTGGAGCAgtaagtggagctcttctgcgtggtgtttggggaataacggtgg

c.540 G>A (p.Thr180=); a common and synonymous variant in the East Asian population (minor allele frequency, 25%).

c.543 G>A (p.Gly181=); a common and synonymous variant in the East Asian population (minor allele frequency, 32%).

**>Exon 5**

ttcgtggatggttcgtcttcctgggaagctgtttctgtcggggccgccgcatccccagcccctgtgactgactctccctctgctctcttccttgcagGGCCTGATCAAGATCCGGGGGGACCGGTGCTGGCGAGACCTCACCTGCATGGACTTCCACTATGAGgtgagcgtgccgtgcagcgtgagggcagggcacccggtctcacgccgccccgttcccactcttggcggggctcagtcctgcacaccctggtgagctgtcgcatcacagggtcacccccattgattcaggacgtcctctccctcccttcacggggtgggtggtcacacgggggcttgctggggctgcggggacggggtctgtggccctccatggtctcgagtgcagctggtttgc

**>Exon 6**

gccaggcgggccggggggatcctgtgtgcagtaggagccggggtcagggggccagggacccacaagcctctccctagccccgccccccgagatcggccaggcacctcacggcccctcctgtccctgcagACCCAGCCGATGCCCAATCCTGTGGCGTACTACCTGCACCACTCACCCTGGTGGTTCCATCGCTTCGAGACGCTCAGCAACCACTTCATCGAGCTCCTGGTGCCCTTCTTCCTCTTCCTCGGCCGGCGGGCGTGCATCATCCACGGGGTGCTGCAGATCCTGTTCCAGgtgagcccgcgccgcccgccctgccccgtgtccacagagacgctggcccgtcgccctatttctggctgccccgct

**>Exon 7**

agagctgggtgcaggttccctcccccctccctgctccaggaagagaggcgccagctgcccaggggccccacacgagcccctgcacgagactcagctgcggcgtctccacagGCCGTCCTCATCGTCAGCGGGAACCTCAGCTTCCTGAACTGGCTGACTATGGTGCCCAGCCTGGCCTGCTTTGATGACGCCACCCTGGGATTCTTGTTCCCCTCTGGGCCAGGCAGCCTGAAGGAC(C/T)GAGTTCTGCAGATGCAGAGGGACATCCGAGGGGCCCGGCCCGAGCCCAGATTCGgtaggtggctcagctgggggcccctgcccaagggcaggagaaagtcgggtggtgccagccccacagcctgcccgtgtgggttggggacgccagggtaggaggagagtcagcgccgtccgcagtgtagcgtgc

c.1024C>T (p.Arg342*); see text.

**>Exon 8**

aggacagctgacccttagggagcagcagctggggtctccccagggacaggccacggggcagctgggagccccacctggaagatgtcactcccctgccagGCTCCGTGGTGCGGCGTGCAGCCAACGTCTCGCTGGGCGTCCTGCTGGCCTGGCTCAGCGTGCCCGTGGTCCTCAACTTGCTGAGCTCCAGGCAGGTCATGAACACCCACTTCAACTCTCTTCACATCGTCAACACTTACGGGGCCTTCGGAAGgtatgagcctggggtccccgtcccccggagctgggtatatggggagactcagccctgaccagccacattcaggtggggttacagtgcaggcccccatgaccccaagtgtcccctgccccaccccagcctgacccaggcacaggccgtgctgtccccactaccgggcctggacacgcaggcctcctcgtc

**>Exon 9**

ccctgaccaggaggcaggtggaggctgtagccctccccctgggccaggatggacagtcggggaaccccccccacccatgcaccccactcggcccagccggtgtgtgacgtgggcctggcctgcctcggtctcccggcagCATCACCAAGGAGCGGGCGGAGGTGATCCTGCAGGGCACAGCCAGCTCCAACGCCAGCGCCCCCGATGCCATGTGGGAGGACTACGAGTTCAAGTGCAAGCCAGGTGACCCCAGCAGACGGCCCTGCCTCATCTCCCCGTACCACTACCGCCTGGACTGGCTGATGTGGTTCGCGGCCTTCCAGgtgggggacggtccctggcgggcgcatggacctgcctgtaccccaccctcttctgcccatagatggcaggtttctagaacgcccactgaaggggctgg

**>Exon 10**

ctcaggagggagtggagggcacagccgagcgtccggaggggctgtgcctggacagcgagtggcacagccgtctccagcccgacctctcctcccagACCTACGAGCACAACGACTGGATCATCCACCTGGCTGGCAAGCTCCTGGCCAGCGACGCCGAGGCCTTGTCCCTGCTGGCACACAACCCCTTCGCGGGCAGGCCCCCGCCCAGgtaggatgccctccctgccgctgctcaggggtctcccccaaatccaggtgtctgctgggatcccaccac

**>Exon 11**

cccggggtctgctgggggtctacctgtgctcagggtgggggctcctcatgcagcccctctgtccgtgggggaccctgtgagcagctgcagctcagcagcaggctgaggagccgggtcgggggccgggagtcccagcaggccctgggctgacatgtgcccccttgcagGTGGGTCCGAGGAGAGCACTACAGGTACAAGTTCAGCCGTCCTGGGGGCAGGCACGCCGCCGAGGGCAAGTGGTGGGTGCGGAAGAGGATCGGAGCCTACTTCCCTCCGCTCAGCCTGGAGGAGCTGAGGCCCTACTTCAGGGACCGTGGGTGGCCTCTGCCCGGGCCCCTCTAGacgtgcaccagaaataaaggcgaagacccagcccctcggcggctcagcaacgtttgcccttccctgcgcccagcccaagctgggcatcg

***LPL* (NM_000237.3)**

**>Exon 1**

gcatcctcattactgtttgctcaacgtttagaagtgaatttaggtccctccccccaacttatgattttatagccaataggtgatgaggtttatttgcatatttccagtcacataagcagccttggcgtgaaaacagtgtcagactcgattccccctcttcctcctcctcaagggaaagctgcccacttctagctgccctgccatcccctttaaagggcgacttgctcagcgccaaaccgcggctccagccctctccagcctccggctcagccggctcatcagtcggtccgcgccttgcagctcctccagagggacgcgccccgagATGGAGAGCAAAGCCCTGCTCGTGCTGACTCTGGCCGTGTGGCTCCAGAGTCTGACCGCCTCCCGCGGAGGGGTGGCCGCCGCCGACCgtaagttttgcgcgcaaactcccctccacctgcagacccggcgggtggccactgccacccgaactgaggatgagaagaaggaagttggaaggggc

**>Exon 2**

tatcattccaatgaataaaatcaagcaaccctccagttaacctcatatccaatttttcctttccagAAAGAAGAGATTTTATCGACATCGAAAGTAAATTTGCCCTAAGGACCCCTGAAGACACAGCTGAGGACACTTGCCACCTCATTCCCGGAGTAGCAGAGTCCGTGGCTACCTGTCATTTCAATCACAGCAGCAAAACCTTCATGGTGATCCATGGCTGGACGgtaagggaggctctttggggaagagtggattggggtggtgaggtatcctgactggcctgcccaattgttggggacccagtgatgggtccgcaccccacatctcacgtggatctccttacacttgaa

**>Exon 3**

gggacagacctgtctctgaacactgttctgttatttgatttttctatctgtgccaatgggtttccaatcaagtttgttttttccatttcatgcaggtgtattgggctgatgtatctatgacaagtggtaggtgggtattttaagaaagcttgtgtcatcatcttcagGTAACAGGAATGTATGAGAGTTGGGTGCCAAAACTTGTGGCCGCCCTGTACAAGAGAGAACCAGACTCCAATGTCATTGTGGTGGACTGGCTGTCACGGGCTCAGGAGCATTACCCAGTGTCCGCGGGCTACACCAAACTGGTGGGACAGGATGTGGCCCGGTTTATCAACTGGATGGAGgtaagactgg

**>Exon 4**

cctgtaacacaaaattaaaataagtagaattagttttcagtatttcctatatttggaaaacaatatttatattcattttgtttcttttagttttatttttggcagaactgtaagcaccttcattttctttttcttccaaagGAGGAGTTTAACTACCCTCTGGACAATGTCCATCTCTTGGGATACAGCCTTGGAGCCCATGCTGCTGGCATTGCAGGAAGTCTGACCAATAAGAAAGTCAACAGAATTACTGgtaagaaagcaatttcgttggtcttatcataagaggtgaaaagactgtcattctgagagagaatcagaacaaattttgttaaatacccacatgtgtggtgttcttcccggagacatgaccagcacttgattatctcatt

**>Exon 5**

aagGCCTCGATCCAGCTGGACCTAACTTTGAGTATGCAGAAGCCCCGAGTCGTCTTTCTCCTGATGATGCAGATTTTGTAGACGTCTTACACACATTCACCAGAGGGTCCCCTGGTCGAAGCATTGGAATCCAGAAACCAGTTGGGCATGTTGACATTTACCCGAATGGAGGTACTTTTCAGCCAGGATGTAACATTGGAGAAGCTATCCGCGTGATTGCAGAGAGAGGACTTGGAGgtaaatattatttagaagcgaattaaatgtgactcttatccttaacccttattgacccaatgtcctactcagtagcttcaaagtatgtagttttcatatacacatttggccaaattatgtttctgaagaattctgcaatgttcagcatgaccaccttagagccaggcagacagccattttatcttttatttactatactgtaggctacactgagcagtgcacttacagtagcaagagaaaaaggtgggatt

**>Exon 6**

ctctaacaccacatctcacctattttagacatgccaaatgaaacactctttgtgaatttctgccgagatacaatcttggtgtctcttttttacccagATGTGGACCAGCTAGTGAAGTGCTCCCACGAGCGCTCCATTCATCTCTTCATCGACTCTCTGTTGAATGAAGAAAATCCAAGTAAGGCCTACAGGTGCAGTTCCAAGGAAGCCTTTGAGAAAGGGCTCTGCTTGAGTTGTAGAAAGAACCGCTGCAACAATCTGGGCTATGAGATCAATAAAGTCAGAGCCAAAAGAAGCAGCAAAATGTACCTGAAGACTCGTTCTCAGATGCCCTACAAAGgtaggctggagactgttgtaaataaggaaaccaaggagtcctatttcatcatgctcactgcatcacatgtactgattctgtccattggaacagagatgatgactggtgttactaaaccctgagccctggtgtttctgtt

**>Exon 7**

TtctgatttgatctccctagcacccctcaaagatggctacttcctaatgctgcttggcaattcagacacatttgggtttttcctatgcatataaccacacttttctgaaagggagtagaattcaaggtctgcattttctaggtatgaacactgtgcatgatgaagtctttccaagccacaccagtggttccatgtgtgtgcacttccggtttgagtgctagtgagatacttctgtggttctgaattgcctgactatttggggttgtgatattttcataaagattgatcaacatgttcgaatttcctccccaacagTCTTCCATTACCAAGTAAAGATTCATTTTTCTGGGACTGAGAGTGAAACCCATACCAATCAGGCCTTTGAGATTTCTCTGTATGGCACCGTGGCCGAGAGTGAGAACATCCCATTCACTCTgtgagtagcacaggggggcggtcatcatggcaccagtccctctcctg

**>Exon 8**

aatttattgcttttttgtttagGCCTGAAGTTTCCACAAATAAGACCTACTCCTTCCTAATTTACACAGAGGTAGATATTGGAGAACTACTCATGTTGAAGCTCAAATGGAAGAGTGATTCATACTTTAGCTGGTCAGACTGGTGGAGCAGTCCCGGCTTCGCCATTCAGAAGATCAGAGTAAAAGCAGGAGAGACTCAGAAAAAgtaattaaatgtatttttcttccttcactttagacccccacctg

**>Exon 9**

aattaactagcttggttgctgaacaccaggttaggctctcaaattaccctctgattctgatgtggcctgagtgtgacagttaattattgggaatatcaaaacaattacccagcatgatcatgtattatttaaacagtcctgacagaactgtacctttgtgaacagtgcttttgattgttctacatggcatattcacatccattttcttccacagGGTGATCTTCTGTTCTAGGGAGAAAGTGTCTCATTTGCAGAAAGGAAAGGCACCTGCGGTATTTGTGAAATGCCATGACAAGTCTCTGAATAAGAAGTCAGGCTGgtgagcattctgggctaaagctgactgggcatcctgagcttgcaccctaagggaggcagcttcatgcattcctcttcaccccatcaccagcagcttgccctgactcatgtgatcaaagcattcaatcagtctttcttagtccttctgcatatgtatcaaatgggtctgttgctttatgcaatacttcctctttttttctttctcctcttgtttctcccagcccggaccttcaacccaggcacacattttaggttttattttactccttgaactacccctgaatcttcacttctccttttttctctactg

***GPIHBP1* (NM_001301772.1)**

**>Exon 1**

ccttcatcccacttaccgcagctccagagccctgcgggaggactcagagtcagggacacagcagcgtccggcgagATGAAGGCGCTCGGGGCTGTCCTGCTTGCCCTCTTGCTGTTCGGGCGGCCAGgtgcggggcaaagggtaaccctgcggtgagggggcagcaacagcagtcctggagcacagggacctccagggacccccagcaggggcttaggaagaaggaggggatgaggctggagtccccagcccaga

**>Exon 2**

gtgttcagggtaggggccctccccagccaccctggggcccgaggatggctggggagggccaaggagttgggggcacgatgcttgcccagagcaggtgtcctccatacccgggtagctgaggcttacaagcatccctgcacggccagGGAGAGGGCAGACACAGCAGGAGGAAGAGGAAGAGGACGAGGACCACGGGCCAGATGACTACGACGAGGAAGATGAGGATGAGGTGGAAGAGGAGGAGACCAACAGGCTCCCTGGTGGCAGGAGCAGAGgtatggccgccccaaccccagagccctgctgcctgatctgcctggagcattctgggcggggctgtgtgatggaagccagcaggccacagtcctgctgtgagcttgcctccagcagagtgggggacac

**>Exon 3 & Exon 4**

caccaggctaggctttgggagcacagctgagaacggggaggtggacagggacgtgggaggagaccctggggggcccggcctcggcctgagcccgccttgtccccagTGCTGCTGCGGTGCTACACCTGCAAGTCCCTGCCCAGGGACGAGCGCTGCAACCTGACGCAGAACTGCTCACATGGCCAGACCTGCACAACCCTCATTGCCCACGGGAACACCGgtaagtgggcgtggggccgcagcacatgcacccccaggcggcgggaaagccaggggcccggaacagagccctgcagagccacctcagagaccccgcccatcctcagcacttgttccccactccccttcccagAGTCAGGCCTCCTGACCACCCACTCCACGTGGTGCACAGACAGCTGCCAGCCCATCACCAAGACGGTGGAGGGGACCCAGgtgaccatgacctgctgccagtccagcctgtgcaatgtcccaccctggcaaagctcccg

***APOC2* (NM_000483.4)**

**>Exon 2**

tgacaccccctcaatgttccaggtctctggacactATGGGCACACGACTCCTCCCAGCTCTGTTTCTTGTCCTCCTGGTATTGGGATTTGgtgagtgtgggcttccggggagggaagccttggggaggggaatgagctccaagcatcttcccagcccaggcccttcttacctctgcctctgccctctcctcttcttccttcctcctttccccctgct

**>Exon 3**

gctgcagccccacgggctctcctgacacactctccccctgcagAGGTCCAGGGGACCCAACAGCCCCAGCAAGATGAGATGCCTAGCCCGACCTTCCTCACCCAGGTGAAGGAATCTCTCTCCAGTTACTGGGAGTCAGCAAAGACAGCCGCCCAGAACCTGTACGAGAAGACATACCTGCCCGCTGTAGATGAGAAACTCAGgtagcacctgcccctggagaaatggggtctggcccataccaccgactgcatccaggacccagaagttcaggcc

**>Exon 4**

accatctgtgctttctccccagGGACTTGTACAGCAAAAGCACAGCAGCCATGAGCACTTACACAGGCATTTTTACTGACCAAGTTCTTTCTGTGCTGAAGGGAGAGGAGTAAcagccagaccccccatcagtggacaaggggagagtcccctactcccctgatcccccaggttcagactgagctcccccttcccagtagctcttgcatcctcctcccaactctagcctgaattcttttcaataaaaaatacaattcaagttgcttctcatggatggcactgcttttctgaggactcaagggccaagatggagg

***APOA5* (NM_001166598.1)**

**>Exon 2 & Exon 3**

ctgtcttctcagagcagataATGGCAAGCATGGCTGCCGTGCTCACCTGGGCTCTGGCTCTTCTTTCAGgtgggtctccgaccctgacttcaacgtgggggtgtgggtggaggctggccagagggccctgtccaccctgggggaggagagcccaggccctgattacctagtccctctccacagCGTTTTCGGCCACCCAGGCACGGAAAGGCTTCTGGGACTACTTCAGCCAGACCAGCGGGGACAAAGGCAGGGTGGAGCAGATCCATCAGCAGAAGATGGCTCGCGAGCCCGCgtgagtgcccaggggaaggggtgtaggcgaagggaggagacagctgggccatgccatgatgacctgcctctgctgcctcaacctctgtggccgctgctgggacagaggaaaggagcggtgcta

**>Exon 4-1**

tttgtatgggcatgtgtttgtgcttcgtgcgtgagttgttactggccagggctaggacaagagccctcgaccctggggccaacgccctgcgtccttggttcccccagaggatcagtgcgcgatgacttggggacaaaggagatgatggaggctagcagtctgacggcctggatatctgtccccttctccagGACCCTGAAAGACAGCCTTGAGCAAGACCTCAACAATATGAACAAGTTCCTGGAAAAGCTGAGGCCTCTGAGTGGGAGCGAGGCTCCTCGGCTCCCACAGGACCCGGTGGGCATGCGGCGGCAGCTGCAGGAGGAGTTGGAGGAGGTGAAGGCTCGCCTCCAGCCCTACATGGCAGAGGCGCACGAGCTGGTGGGCTGGAATTTGGAGGGCTTGCGGCAGCAACTGAAGCCCTA

**>Exon 4-2**

CTACACGATGGATCTGATGGAGCAGGTGGCCCTGCGCGTGCAGGAGCTGCAGGAGCAGTTGCGCGTGGTGGGGGAAGACACCAAGGCCCAGTTGCTGGGGGGCGTGGACGAGGCTTGGGCTTTGCTGCAGGGACTGCAGAGCCGCGTGGTGCACCACACCGGCCGCTTCAAAGAGCTCTTCCACCCATACGCCGAGAGCCTGGTGAGCGGCATCGGGCGCCACGTGCAGGAGCTGCACCGCAGTGTGGCTCCGCACGCCCCCGCCAGCCCCGCGCGCCTCAGTCGCTGCGTGCAGGTGCTCTCCCGGAAGCTCACGCTCAAGGCCAAGGCCCTGCACGCACGCATCCAGCAGAACCTGGACCAGCTGCGCGAAGAGCTCAGCAGAGCCTTTGCAGGCACTGGGACTGAGGAAGGGGCCGGCCCGGACCCCCAGATGCTCTCCGAGGAGGTGCGCCAGCGACTTCAGGCTTTCCGCCAGGACACCTACCTGCAGATAGCTGCCTTCACTCGC

**>Exon 4-3**

AGAGCTCAGCAGAGCCTTTGCAGGCACTGGGACTGAGGAAGGGGCCGGCCCGGACCCCCAGATGCTCTCCGAGGAGGTGCGCCAGCGACTTCAGGCTTTCCGCCAGGACACCTACCTGCAGATAGCTGCCTTCACTCGCGCCATCGACCAGGAGACTGAGGAGGTCCAGCAGCAGCTGGCGCCACCTCCACCAGGCCACAGTGCCTTCGCCCCAGAGTTTCAACAAACAGACAGTGGCAAGGTTCTGAGCAAGCTGCAGGCCCGTCTGGATGACCTGTGGGAAGACATCACTCACAGCCTTCATGACCAGGGCCACAGCCATCTGGGGGACCCCTGAggatctacctgcccaggcccattcccagctccttgtctggggagccttggctctgagcctctagcatggttcagtccttgaaagtggcctgtt
